# Special Issue of the 1st International Applied Bioinformatics Conference (iABC'21)

**DOI:** 10.1515/jib-2021-0042

**Published:** 2021-12-16

**Authors:** Jens Allmer, Mourad Elloumi, Matteo Comin, Ralf Hofestädt

**Affiliations:** Medical Informatics and Bioinformatics, Hochschule Ruhr West, Institute for Measurement Engineering and Sensor Technology, Mülheim an der Ruhr, Germany; Faculty of Computing and Information Technology, The University of Bisha, Bisha, Saudi Arabia; Department of Information Engineering, University of Padua, Padua, Italy; Technical Faculty, AG Bioinformatics and Medical Informatics, Bielefeld University, Bielefeld, Germany

Diseases can be tied to changes at the molecular level within affected cells. This can be concerning transcription, translation, or any other mechanism involved in gene expression, such as post-transcriptional regulation. Instrumentation for the measurement of such molecular changes is readily available and produces large amounts of data. For example, DNA and RNA sequencing, as well as protein quantitation, and sequencing can be achieved via next-generation sequencing and mass spectrometry, respectively. One current challenge is the analysis and integration of the resulting heterogeneous and large datasets.

Bioinformatics is the field of study which produces algorithms and integrative approaches to attempt such data analyses. The primary aim in algorithmic bioinformatics is, however, the development of algorithms and not their application. Typically, novel algorithms are introduced with a proof of principle, and they are applied to some data for that purpose, but usually not comprehensively. Their data might slightly differ from the proof of principle, inducing further data analysis challenges. Additionally, applying such algorithms to their data may be involved for researchers from the biomedical domain.

The 1st International Applied Bioinformatics Conference was conceived to bring together representatives from all research fields involved to increase knowledge transfer. First planned for 2020 and then deferred to 2021 due to the pandemic caused by the Coronavirus [1], the conference was held online. Despite the virtual nature of the conference, attention was great. We received many good manuscripts and invited a few to submit their full versions to this special issue. The range of topics was extensive, but many submissions concerned the interface of bioinformatics and its application. The selected papers for this special issue also discuss various topics such as sequence alignment and gene network reconstruction.

The first paper in this special issue concerns a challenging issue in bioinformatics, the usage of pan-genomes instead of single reference genomes and offers a fast variation-aware read mapping algorithm [2]. Mapping is also vital to investigate gene expression, which is essential for the second manuscript. It discusses how microRNA and mRNA expression profiles can be investigated [3]. From this, modular networks are inferred, describing post-transcriptional regulatory networks. Such networks are challenging to visualize, which is the focus of the third paper [4]. The work summarizes the state-of-the-art in bicluster visualization and is also based on gene expression data. Next, we move from transcriptomics to metabolomics. A disparity filter was applied to perform network analysis for colorectal cancer as a proof of principle [5]. The final two manuscripts focus more on practical application in cancer. First, the prostate, ovary, testes, and embryo gene family paralogs (POTEE) are investigated in silico based on epigenetics in ovarian cancer [6]. Secondly, gene networks were reconstructed for glioblastoma [7]. In summary, this special issue encompasses novel algorithms, algorithm transfer from a different domain, and the application of bioinformatics tools on the analysis of several cancers.

We hope that the 2nd International Applied Bioinformatics Conference will be held in hybrid (in presence, but including live streams).
